# Formamidinium Lead Halide Perovskite Nanocomposite Scintillators

**DOI:** 10.3390/nano12132141

**Published:** 2022-06-22

**Authors:** Isabel H. B. Braddock, Maya Al Sid Cheikh, Joydip Ghosh, Roma E. Mulholland, Joseph G. O’Neill, Vlad Stolojan, Carol Crean, Stephen J. Sweeney, Paul J. Sellin

**Affiliations:** 1 Department of Physics, University of Surrey, Guildford GU2 7XH, UK; i.braddock@surrey.ac.uk (I.H.B.B.); j.ghosh@surrey.ac.uk (J.G.); s.sweeney@surrey.ac.uk (S.J.S.); 2 Department of Chemistry, University of Surrey, Guildford GU2 7XH, UK; m.alsidcheikh@surrey.ac.uk (M.A.S.C.); rm00849@surrey.ac.uk (R.E.M.); c.crean@surrey.ac.uk (C.C.); 3 Advanced Technology Institute, University of Surrey, Guildford GU2 7XH, UK; v.stolojan@surrey.ac.uk

**Keywords:** perovskite nanocrystal, formamidinium lead halide (FAPbX_3_), plastic scintillator, nanocomposite scintillator, X-ray imaging

## Abstract

While there is great demand for effective, affordable radiation detectors in various applications, many commonly used scintillators have major drawbacks. Conventional inorganic scintillators have a fixed emission wavelength and require expensive, high-temperature synthesis; plastic scintillators, while fast, inexpensive, and robust, have low atomic numbers, limiting their X-ray stopping power. Formamidinium lead halide perovskite nanocrystals show promise as scintillators due to their high X-ray attenuation coefficient and bright luminescence. Here, we used a room-temperature, solution-growth method to produce mixed-halide FAPbX${}_{3}$ (X = Cl, Br) nanocrystals with emission wavelengths that can be varied between 403 and 531 nm via adjustments to the halide ratio. The substitution of bromine for increasing amounts of chlorine resulted in violet emission with faster lifetimes, while larger proportions of bromine resulted in green emission with increased luminescence intensity. By loading FAPbBr${}_{3}$ nanocrystals into a PVT-based plastic scintillator matrix, we produced 1 mm-thick nanocomposite scintillators, which have brighter luminescence than the PVT-based plastic scintillator alone. While nanocomposites such as these are often opaque due to optical scattering from aggregates of the nanoparticles, we used a surface modification technique to improve transmission through the composites. A composite of FAPbBr${}_{3}$ nanocrystals encapsulated in inert PMMA produced even stronger luminescence, with intensity 3.8× greater than a comparative FAPbBr${}_{3}$/plastic scintillator composite. However, the luminescence decay time of the FAPbBr${}_{3}$/PMMA composite was more than 3× slower than that of the FAPbBr${}_{3}$/plastic scintillator composite. We also demonstrate the potential of these lead halide perovskite nanocomposite scintillators for low-cost X-ray imaging applications.

## 1. Introduction

The applications of radiation detectors are extensive and varied, ranging from medical imaging to high-energy physics research and nuclear security [1,2].

Scintillating materials convert high-energy X-ray or gamma-ray photons into many visible-light photons, which can then be detected via a photomultiplier tube or similar photodetector. An effective scintillator should have a high light yield, fast timing, and a high attenuation coefficient. In order to be of practical use, it should also be manufactured at a reasonably low cost. Plastic scintillators are robust and cost-effective and have very fast timings. However, as the absorption coefficient in the photoelectric effect is proportional to $Z^{n},4<n<5$, their ability to stop X-rays is limited [2]. The production of a nanocomposite scintillator, in which high-Z particles are added to a plastic scintillator in order to increase the overall atomic number of the scintillator, is therefore an area of research with particular potential [3].

As well as widespread research into their use in solar cells [4,5] and various optoelectronic devices such as LEDs and lasers [6,7], lead halide perovskites (LHPs) have also shown promise for radiation detection applications. While single-crystal LHPs have shown a high light yield only at low temperatures [8,9], the development of nanocrystalline LHPs (beginning in 2014 [10]) has led to the rapid development of nanocrystal LHP scintillators: as colloidal dispersions [11,12,13,14], thin films [15,16,17], or encapsulated in inert plastics [18,19]. However, the interaction of LHP nanocrystals with commercial plastic scintillators within a nanocomposite has not previously been reported. Due to the high atomic number of lead, LHPs have high attenuation coefficients. Their luminescence wavelength is adjustable throughout the visible region via the selection of different combinations of halide ions, and hence may be chosen to suit the application. Unlike conventional inorganic scintillator crystals, nanocrystal LHPs are solution-processable at room temperature, which allows for low-cost manufacture. They have typically been found to have high photoluminescence quantum yields [20,21,22] and short photoluminescence decay times [23,24].

A significant challenge in producing uniform, transparent nanocomposite materials comes from the aggregation of nanoparticles during mixing. This tendency to agglomerate is caused by van der Waals interactions, as well as by complex enthalpic and entropic effects, which are influenced by the high surface energy of the nanoparticles and by the excluded volume effect from the polymer molecules [3,25,26,27]. As transmittance loss due to light scattering from nanocrystals is strongly dependent on particle size [28], the formation of aggregates has a severe negative effect on the transparency of the composite, reducing the efficiency of the scintillator. However, as interactions between the nanoparticles and polymer are mediated by surface ligands, it is possible to influence the distribution of nanocrystals within the composite via surface modification of the nanocrystal. To this end, bis(2-(methacryloyloxy)ethyl) phosphate (BMEP) can be used to couple nanocrystals with the polymer matrix during polymerisation. BMEP has previously been reported as a surface ligand in nanocomposite scintillators that contained Gd${}_{2}$O${}_{3}$ [29], HfO${}_{2}$ [30], YbF${}_{3}$ [31], Cd${}_{x}$Zn${}_{1-x}$/ZnS core/shell quantum dots [32] and, recently, the all-inorganic LHP CsPbBr${}_{3}$ [33], but it has not previously been tested with FAPbX${}_{3}$ perovskites. In the case of Cd${}_{x}$Zn${}_{1-x}$/ZnS, composites with up to 60% loading of nanoparticles were produced, with high transparency facilitated by the BMEP ligand [32].

In this work, we report the production of mixed-halide formamidinium lead halide perovskite nanocrystals via a simple, room-temperature synthesis. These nanocrystals exhibit fast lifetimes and bright emission with tunable wavelength between 403 and 531 nm. The scintillation properties of FAPbBr${}_{3}$ have previously been reported only for colloidal nanocrystals [14] and for thin films of nanocrystals [34], while here, we produced nanocomposite scintillators consisting of green-emitting FAPbBr${}_{3}$ nanocrystals within either an EJ-290 plastic scintillator matrix or a PMMA matrix and demonstrate an improvement in transparency via surface modification using BMEP. The nanocomposite scintillators (particularly those with a PMMA matrix) show brighter luminescence than EJ-290 plastic scintillator alone; those with an EJ-290 matrix show faster lifetimes than FAPbBr${}_{3}$ nanocrystals encapsulated in PMMA. We also demonstrate the use of the nanocomposite scintillators for X-ray imaging.

## 2. Results and Discussion

### 2.1. Optical Properties of Perovskite Nanocrystals

Perovskites are a class of materials that have great diversity in their potential components. They are characterised by their ABX${}_{3}$ structure, in which the *B* cation sits at the centre of an octahedron of *X* anions, with the *A* cations occupying the interstitial spaces between these octahedra. Lead halide perovskites are specifically those perovskites in which the *B* cation is lead (which provides a benefit in terms of interaction probability, due to its high atomic number), and the *X* cation is bromine, chlorine, iodine, or some combination of these. The *A* cation is commonly either caesium or an organic ion such as methyl ammonium (CH${}_{3}$NH${}_{3}$) or formamidinium (CH${}_{5}$N${}_{2}$). Here, formamidinium was chosen as the *A* cation.

Formamidinium lead halide nanocrystals (FAPbX${}_{3}$, X = Br, Cl) were synthesised using a ligand-assisted reprecipitation method, which was conducted at room temperature. The ligands used were octylamine, which limits the growth of the nanocrystal, and oleic acid, which is necessary for stability [10,35]. The synthesis was based on that of Perumal et al. [36] and is described further in the Methods Section.

**Figure 1:**
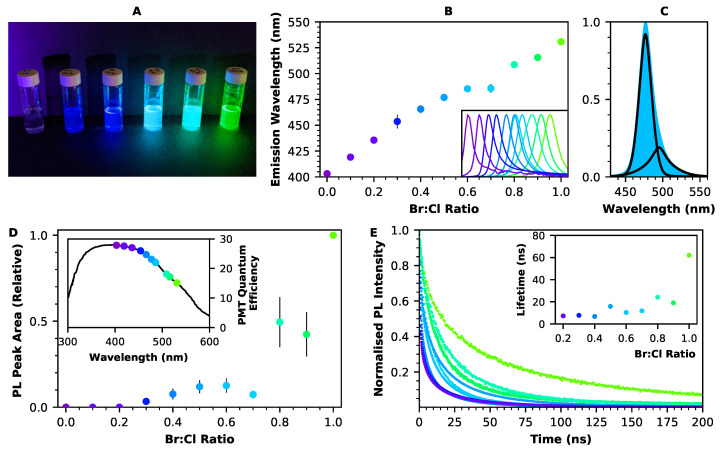
Optical characterisation for FAPbBr${}_{3x}$Cl${}_{3(1-x)}$ nanocrystals. (**A**) Photograph of dispersed FAPbBr${}_{3x}$Cl${}_{3(1-x)}$ nanocrystals under UV light; from left to right: x = 0, 0.2, 0.4, 0.6, 0.8, 1 (**B**) The peak emission wavelength of FAPbBr${}_{3x}$Cl${}_{3(1-x)}$ nanocrystals is dependent on the halide content of the nanocrystals. Inset shows the normalised photoluminescence spectrum for each sample. (**C**) Photoluminescence peaks from FAPbBr${}_{3x}$Cl${}_{3(1-x)}$ nanocrystals can be fitted with two components. Fitting is shown here for FAPbBr${}_{1.5}$Cl${}_{1.5}$. (**D**) The area under the photoluminescence peaks of the mixed-halide nanocrystals is strongly influenced by the halide content, with more bromine giving a brighter nanocrystal. Inset shows the quantum efficiency of ET Enterprises 9256B PMT, marked with the wavelength of each perovskite nanocrystal dispersion. (**E**) Photoluminescence decay spectra for mixed-halide perovskite nanocrystals in PMMA. Inset shows the average lifetime for each sample, as obtained from a two-exponential fit.

The emission wavelength of these perovskite nanocrystals may be adjusted throughout the entire visible spectrum via small changes to the composition. This wavelength tuning is made possible by the electronic structure of the lead halide perovskite, in which both the conduction and valence bands are formed from contributions from both lead and halide orbitals [37,38]. To this end, FAPbCl${}_{3}$ and FAPbBr${}_{3}$ nanocrystals were produced, as well as mixed-halide nanocrystals with varying ratios of bromine to chlorine. Figure 1A shows dispersions of these nanocrystals in toluene, illuminated by a UV torch, demonstrating their luminescence in a range of colours, from purple to green. If necessary, emission wavelengths in red could also be produced, with iodine as the *X* cation (see, e.g., [39]).

Figure 1B shows emission wavelengths for the FAPbX${}_{3}$ nanocrystal dispersions, as measured from their room-temperature photoluminescence spectra (shown in the inset). These wavelengths range from 403 nm (for FAPbCl${}_{3}$) to 531 nm (for FAPbBr${}_{3}$) and have a strong linear dependency on the halide ratio, meaning that is possible to fine-tune the emission wavelength via further small adjustments to the LHP composition. Wavelengths are also dependent on the size of the nanocrystal, leading to some variation between samples. This is an effect of weak quantum confinement as the nanocrystal size moves towards the Bohr exciton radius (approximately 3.5 nm for FAPbBr${}_{3}$ [40]). The effect of nanocrystal size on the emission wavelength of FAPbBr${}_{3}$ is shown in the Supporting Information, Figure S1. The room-temperature photoluminescence peaks for these FAPbBr${}_{3x}$Cl${}_{3(1-x)}$ nanocrystals are relatively narrow: for example, the FWHM for the FAPbBr${}_{3}$ nanocrystals is 23.7±0.5 nm. Within the emission peak for each sample, a low-energy tail is visible, meaning that two components are necessary for peak fitting. Figure 1C shows an example of this fitting, using a pseudo-Voigt profile as described in the Methods Section.

One advantage of the continuous range of emission wavelengths seen in FAPbBr${}_{3x}$Cl${}_{3(1-x)}$ is that the nanocrystal luminescence may be tuned so as to match the wavelength at which the quantum efficiency of a particular detector is maximised. For a photomultiplier tube, this wavelength might typically be around 400 nm, while a CCD or CMOS image sensor may have a much broader wavelength range. The inset of Figure 1D shows the emission wavelengths of each of the mixed-halide FAPbBr${}_{3x}$Cl${}_{3(1-x)}$ nanocrystals marked against the quantum efficiency of an ET Enterprises 9256B PMT. In the case of the purple-emitting FAPbCl${}_{3}$, the quantum efficiency of the photomultiplier tube is approximately doubled compared to that which is obtained at the longer emission wavelength of FAPbBr${}_{3}$. However, for those samples with a higher Cl concentration, there is a significant reduction in light intensity (Figure 1D), which tends to negate their efficacy for detector applications.

Figure 1E shows time-resolved photoluminescence for the FAPbX${}_{3}$ nanocrystals embedded in PMMA discs. The luminescence decays were fitted with a two-exponential fit, and the average time constant for each type of LHP nanocrystal is shown in the inset of Figure 1E. FAPbBr${}_{3}$ had a fast component of 16.9 ns, which contributed 56% of the emission, and a slow component of 119.7 ns, which contributed the remaining 44%, leading to an average decay time of 62.0 ns. Samples emitting at shorter wavelengths, corresponding to wider band gaps, were found to have faster decay times, which is consistent with previous literature [40,41]; a full table of time constants can be found in the Supporting Information, Table S1. The fast component of the decay is typically associated with traps on the nanocrystal surface, while the slower component is attributed to recombination inside the nanocrystal [36,42].

Overall, due to its bright luminescence, FAPbBr${}_{3}$ was chosen for inclusion in the nanocomposite scintillators.

### 2.2. Optimising Transmission through Nanocomposite Scintillators

In order to optimise the performance of a nanocomposite scintillator, the effective atomic number must be increased as much as possible. However, scattering of light from the embedded nanocrystals may compromise the light yield in composites with higher loadings. Transmission losses due to scattering are greater in the case of a thicker composite, a higher volume loading of nanocrystals, a mismatch between the refractive indices of particle and matrix, and a larger particle size. However, it is important to maintain a relatively thick scintillator, with the greatest possible loading of nanocrystals so as to maximise the attenuation coefficient of the scintillator. Therefore, an appropriately small size of nanocrystal must be obtained for the composite to be useful.

**Figure 2:**
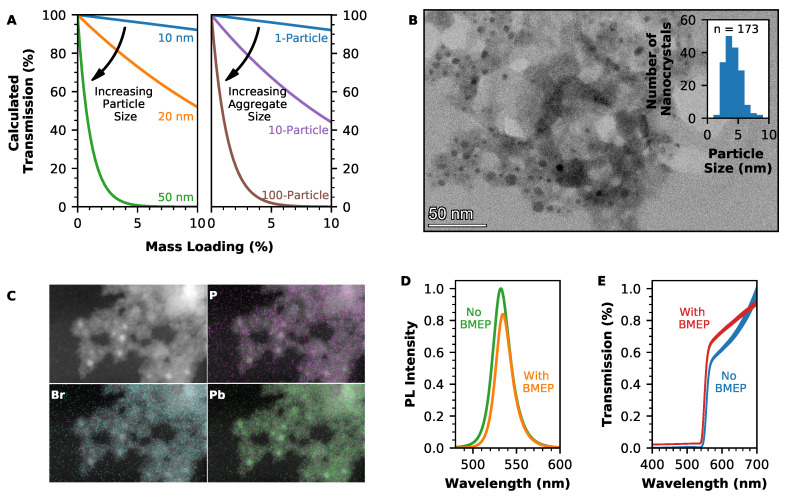
(**A**) Calculated effect of Mie scattering on transmission through a 1 mm-thick FAPbBr${}_{3}$/PVT composite as the nanocrystal loading is increased, either for various sizes of nanocrystal (left) or for aggregates of 10 nm particles (right). (**B**) High-resolution TEM image, showing FAPbBr${}_{3}$ nanocrystals as dark features surrounded by the BMEP ligand. Inset shows the distribution of nanocrystal sizes, as measured from 173 nanocrystals. (**C**) High-angle annular dark field (HAADF) and EDS maps performed in scanning TEM mode, showing individual elemental maps for phosphorus (top right), bromine (bottom left), and lead (bottom right) in FAPbBr${}_{3}$ nanocrystals with the BMEP surface ligand, overlaid on the HAADF image of of the nanocrystals (top left). (**D**) Photoluminescence of FAPbBr${}_{3}$ nanocrystal dispersions either with (orange) or without (green) the BMEP ligand, showing a reduction in luminescence intensity when the BMEP ligand is used. (**E**) Transmission through the FAPbBr${}_{3}$/EJ-290 nanocomposite scintillator either with (red) or without (blue) the BMEP ligand, showing improved transmission when the BMEP ligand is used.

For a 1 mm-thick disc of nanocomposite, which contains FAPbBr${}_{3}$ perovskite nanocrystals in an EJ-290 plastic scintillator matrix, an estimate of the expected transmission after scattering is shown in Figure 2A. These results were obtained using calculations based on Mie scattering theory and assume a monodisperse particle size distribution with negligible instances of multiple scattering [28]. While Rayleigh scattering is often used to describe scattering in nanocomposites such as these, this is an approximation of Mie scattering, which is only valid for particles that are small compared to the wavelength of the light that is used to illuminate them. Mie scattering theory can be applied to particles of any size and is therefore particularly important when considering the case in which the scattering centres are aggregates of particles.

As Figure 2A shows, when nanocrystals of FAPbBr${}_{3}$ are homogeneously distributed through the plastic scintillator matrix, a particle size of 10 nm is sufficiently small that transmission is only impacted at higher loadings. However, when those 10 nm particles have formed aggregates within the composite, the transmission begins to drop off very rapidly: if a typical aggregate consists of 100 particles, then the transmission through the composite will be similar to that for a composite containing individually dispersed particles that are 50 nm in diameter. Therefore, it is not sufficient only to reduce the size of the nanocrystals; the issue of aggregation must be addressed simultaneously.

Prior to the production of nanocomposites, the FAPbBr${}_{3}$ nanocrystals were modified by partial ligand exchange with the bifunctional ligand BMEP. The P-OH head of this ligand binds strongly to the surface of the nanocrystals, while the vinyl group of the ligand co-polymerises with the PVT in the plastic scintillator matrix, preventing aggregation of the nanocrystals during polymerisation.

A TEM image of FAPbBr${}_{3}$ nanocrystals is shown in Figure 2B, with the distribution of nanocrystal sizes shown in the inset. The nanocrystals were spherical, with a mean diameter of 4 nm. Compared to the Bohr radius for FAPbBr${}_{3}$, this is sufficiently small for the nanocrystals to exhibit quantum confinement effects. However, this 4 nm diameter does not include the long-chain oleic acid, octylamine, or BMEP ligands attached to the nanocrystal surface. The hydrodynamic radius of the FAPbBr${}_{3}$ nanocrystals was measured through dynamic light scattering to be (19 ± 4) nm (see Figure S2 in the Supporting Information). Within the TEM image, the BMEP ligand can be seen as a lighter halo surrounding the nanocrystal core (a TEM image of nanocrystals without BMEP modification is shown in the Supporting Information, Figure S3).

Figure 2C shows the EDS mapping of elements in BMEP-modified nanocrystals. The location of the FAPbBr${}_{3}$ nanocrystals is shown by the lead and bromine maps; the correlation of phosphorus from the BMEP ligand around these elements indicates successful attachment of the ligand to the nanocrystals. As the original oleic acid ligands are only partially replaced by BMEP, the FAPbBr${}_{3}$ nanocrystal is not destabilised by the ligand exchange. However, the addition of the BMEP ligand does have the effect of reducing the luminescence intensity of the nanocrystal. Figure 2D shows photoluminescence spectra for toluene dispersions of FAPbBr${}_{3}$ nanocrystals either with or without the BMEP modification. In the case where the partial ligand exchange has taken place, the area under the peak is reduced by 23%. A similar effect was noted previously by Liu et al. for BMEP modification of Cd${}_{x}$Z${}_{1-x}$S/ZnS quantum dots: a reduction in photoluminescence quantum yield from 83.8% to 77.4% was reported in that case [32]. For the BMEP ligand to be a worthwhile addition to a nanocomposite scintillator, luminescence detected from the nanocomposite must be greater with the ligand than without. Therefore, the effect of the ligand on increasing the transmission of light through the composite must be sufficient that it negates this reduction in intensity, leading to an improved light yield from the scintillator overall.

Nanocomposite discs were produced using BMEP-modified FAPbBr${}_{3}$ nanocrystals, with the PVT-based plastic scintillator EJ-290 as the matrix. The addition of the BMEP ligand successfully reduced the aggregation of nanocrystals (see also Figure S4, Supporting Information), leading to an improvement in transmission through the nanocomposite. Figure 2E compares transmission through a 1.2 mm-thick nanocomposite containing BMEP-modified FAPbBr${}_{3}$ nanocrystals and a 0.9 mm-thick nanocomposite containing an equivalent loading of FAPbBr${}_{3}$ nanocrystals without BMEP modification. At a 600 nm wavelength, transmission through the composite with the BMEP ligand is 18% greater than for the composite without the ligand, despite the greater thickness of the composite containing BMEP-modified nanocrystals.

Despite the BMEP modification, there is a low level of transmission through the composites overall, suggesting that factors other than aggregation may be responsible. A likely cause is re-absorption: the Stokes shift of the FAPbBr${}_{3}$ nanocrystals is very small, at 50 meV (Figure S5 in the Supporting Information), meaning that there is a high probability that any photon emitted by the nanocrystals will be reabsorbed as it passes through the composite. The overall quantum yield of the scintillator is reduced with each instance of photon re-absorption due to the non-zero probability of non-radiative recombination, leading to a reduction in the light transmitted through the composite.

### 2.3. Performance of Nanocomposite Scintillators

**Figure 3:**
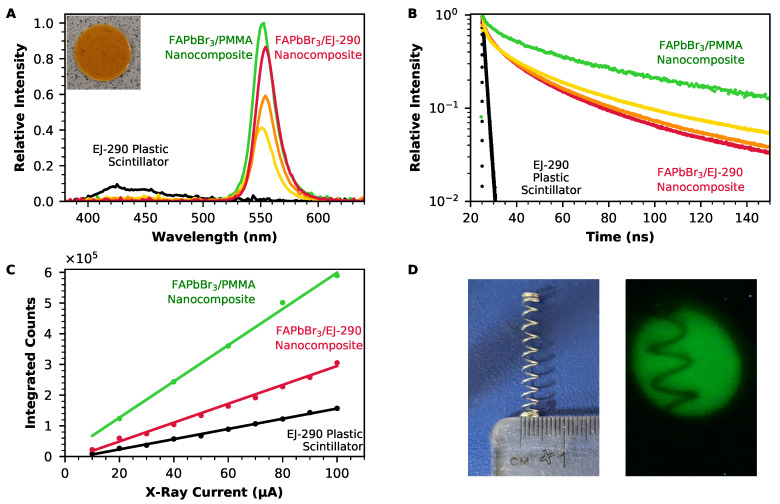
(**A**) Radioluminescence spectra for the FAPbBr${}_{3}$/PMMA nanocomposite with 1.8 wt% loading of FAPbBr${}_{3}$ nanocrystals (green), FAPbBr${}_{3}$/EJ-290 nanocomposite scintillators with 0.4 wt% (yellow), 0.9 wt% (orange), and 1.8 wt% (red) loading of FAPbBr${}_{3}$ nanocrystals and for a 1 mm-thick disc of EJ-290 plastic scintillator (black). (**B**) Photoluminescence decays for the 1.8 wt% loading FAPbBr${}_{3}$/PMMA nanocomposite (green), FAPbBr${}_{3}$/EJ-290 nanocomposite scintillators of various loadings (0.4%/0.9%/1.8%, yellow/orange/red), and the EJ-290 plastic scintillator (black). (**C**) Radioluminescence intensity as a function of the dose rate for the FAPbBr${}_{3}$/PMMA nanocomposite with 1.8 wt% loading (green), FAPbBr${}_{3}$/EJ-290 nanocomposite with 1.8 wt% loading (red), and EJ-290 plastic scintillator (black). (**D**) Photograph (left) and X-ray image (right) of metal spring, acquired using the 1.8 wt% loading FAPbBr${}_{3}$/PMMA nanocomposite scintillator.

The performance of the perovskite nanocomposite scintillators is demonstrated in Figure 3. These composites have 0.4 wt%, 0.9 wt%, or 1.8 wt% loading of BMEP-modified FAPbBr${}_{3}$ nanocrystals in EJ-290; they are also compared to a composite that has 1.8 wt% loading of FAPbBr${}_{3}$ in PMMA (without BMEP modification). The lead content of the nanocomposites was confirmed using microwave plasma atomic emission spectrometry of the nanocomposite samples (see Supporting Information, Figure S6 and Table S2 for more information).

Figure 3A shows radioluminescence spectra for the FAPbBr${}_{3}$/EJ-290 and FAPbBr${}_{3}$/PMMA composites, as well as for an equally sized disc of EJ-290 plastic scintillator. The spectrum recorded from EJ-290 has multiple luminescence peaks between 400 nm and 600 nm, with the brightest occurring at 423 nm and 445 nm. However, these peaks are suppressed in each of the spectra from the nanocomposite scintillators. Instead, a single peak is visible in each case, due to the FAPbBr${}_{3}$ perovskite nanocrystals. The intensity of this peak increases as the loading of FAPbBr${}_{3}$ is increased. The suppression of the higher-energy plastic scintillator luminescence indicates energy transfer from the plastic scintillator to the perovskite nanocrystals, either through absorption of the plastic scintillator luminescence by the nanocrystals or through a non-radiative energy transfer mechanism such as Förster resonant energy transfer (FRET) [43].

The peak emission wavelengths for the FAPbBr${}_{3}$/EJ-290 nanocomposites with 0.4%, 0.9%, and 1.8% loading occur at 551 nm, 554 nm, and 554 nm, respectively. These emission wavelengths are red-shifted from the 531 nm emission recorded for the same nanocrystals in the toluene dispersion, particularly for the two composites with a higher loading. This wavelength shift is consistent with self-absorption in the composite, which occurs due to the small Stokes shift of FAPbBr${}_{3}$. As the absorption curve of the perovskite overlaps the emission peak at higher energies, only the long-wavelength tail of the initial luminescence is transmitted through the scintillator.

The time response of the nanocomposite scintillators is shown in Figure 3B. For each of the FAPbBr${}_{3}$/EJ-290 composites, the decay can be fit with a three-part exponential, corresponding to two components from the nanocrystals and one from the plastic scintillator. The time constants obtained from these exponential fits are shown in Table 1. For each of the nanocomposites, the average decay time is slower than the very fast decay obtained from the EJ-290 plastic scintillator without nanocrystal loading. The longest decay time was obtained from the FAPbBr${}_{3}$/PMMA nanocomposite, while for the FAPbBr${}_{3}$/EJ-290 nanocomposite, decay times were faster when the composite contained a higher loading of the FAPbBr${}_{3}$ perovskite nanocrystals. These faster overall decay times are due to a decrease in the two components of the time constant that are linked to the nanocrystals and are likely to be related to energy transfer from the plastic scintillator matrix to the perovskite nanocrystals. One indication of the presence of an energy transfer process between a donor and an acceptor species is an increase in the lifetime of the acceptor, occurring when the concentration of the donor is increased [44]. In this case, as is seen from the radioluminescence spectra shown in Figure 3A, energy transfer within the composite occurs from the EJ-290 (donor) to the FAPbBr${}_{3}$ nanocrystals (acceptor). As the loading of the scintillator decreases, the relative concentration of the donor (EJ-290) increases, which leads to an increase in the lifetime of the acceptor (FAPbBr${}_{3}$). This explains why adding more of the FAPbBr${}_{3}$ results in a decrease in the lifetime of the composite overall, despite the slower decay time of the FAPbBr${}_{3}$ nanocrystals compared to the EJ-290 plastic scintillator.

Figure 3C compares the sensitivity of the FAPbBr${}_{3}$/EJ-290 nanocomposite scintillator, FAPbBr${}_{3}$/PMMA nanocomposite with equivalent mass loading of FAPbBr${}_{3}$ nanocrystals, and EJ-290 plastic scintillator. As the X-ray tube current (and therefore, the dose rate) was incrementally increased, the radioluminescence spectra were integrated over all wavelengths to give the total amount of light emitted by the scintillator at each value of the dose rate. All three samples had a linear response to increasing X-ray dose rate. The performance of both the FAPbBr${}_{3}$/EJ-290 and FAPbBr${}_{3}$/PMMA nanocomposites surpassed that of the EJ-290 plastic scintillator: at the maximum dose rate (0.68 Gy/s), the number of counts recorded for the FAPbBr${}_{3}$/EJ-290 composite was 1.9× that of the EJ-290, while the number of counts recorded for the FAPbBr${}_{3}$/PMMA composite with equivalent loading was significantly greater, at 3.8× that of the EJ-290.

The suitability of the nanocomposite scintillators for X-ray imaging was assessed by acquiring images using the FAPbBr${}_{3}$ nanocomposite scintillators with an 80 kV X-ray source and a digital webcam. The results of this are presented in Figure 3D, which shows the clear X-ray image obtained using the FAPbBr${}_{3}$/PMMA nanocomposite, of a small metal spring with a 4 mm diameter.

## 3. Conclusions

Mixed-halide FAPbBr${}_{x}$Cl${}_{3(1-x)}$ perovskite nanocrystals were made using a room-temperature solution growth method. These nanocrystals had emission wavelengths between 405 nm (for FAPbCl${}_{3}$) and 531 nm (for FAPbBr${}_{3}$) and photoluminescence lifetimes that decreased as more chlorine was added. Although shorter emission wavelengths and faster luminescence decay times would both be of benefit to scintillators, the luminescence recorded from FAPbBr${}_{3}$ was approximately 10,000× more intense from that recorded from FAPbCl${}_{3}$, and therefore, FAPbBr${}_{3}$ nanocrystals were chosen to produce nanocomposite scintillators.

The 1 mm-thick nanocomposite scintillators were produced using FAPbBr${}_{3}$ nanocrystals and EJ-290 plastic scintillator, with loadings up to 1.8%. Nanocrystals were modified using BMEP to prevent aggregation during polymerisation of the composite. While this modification reduced the scattering of light passing through the composite, transmission through the composite was also adversely affected by light reabsorption caused by the small Stokes shift of the perovskite nanocrystals. The addition of the BMEP ligand to the nanocrystals also reduced the intensity of the luminescence detected from the FAPbBr${}_{3}$ nanocrystals.

The increased loading of FAPbBr${}_{3}$ nanocrystals within the FAPbBr${}_{3}$/EJ-290 composite led to brighter scintillation from the composites, surpassing the performance of the EJ-290 plastic scintillator alone. The performance of a FAPbBr${}_{3}$/PMMA composite was also compared: although this composite had a slower time response than the FAPbBr${}_{3}$/EJ-290 system, it also produced significantly brighter emission overall. Therefore, while the BMEP ligand had some benefit in increasing transmission through the nanocomposite, the performance of the BMEP-modified FAPbBr${}_{3}$/EJ-290 system was surpassed by a simpler FAPbBr${}_{3}$/PMMA system, which did not use the BMEP ligand. The suitability of these nanocomposites for low-cost X-ray imaging was demonstrated. Further improvements to these nanocomposite scintillators could be made by incorporating a wavelength-shifting dye, which would increase Stokes shift and prevent self-absorption. This would allow for the production of composites with increased nanocrystal loadings, while retaining the luminescence intensity of the scintillator.

## 4. Materials and Methods

### 4.1. Materials

Lead bromide (PbBr${}_{2},\geq$98%), dimethylformamide (DMF, 99.8%), formamidinium chloride (98% Purity), octylamine (99%), Bis[2-(methacryloyloxy)ethyl] phosphate (BMEP), and poly(methyl methacrylate) (PMMA, average Mw 350,000) were purchased from Sigma Aldrich. Toluene (≥99.8%) and oleic acid (<70%) were purchased from Fisher Scientific. Formamidinium bromide (>99.5%) was purchased from Osilla (Sheffield, UK). Lead chloride (PbCl${}_{2}$, 99%) was purchased from Acros Organics. EJ-290 Plastic Scintillator Casting Resin was purchased from Eljen Technology (Sweetwater, TX, USA).

### 4.2. Perovskite Nanocrystal Synthesis

The synthesis method used was similar to that of Perumal et al. [36]. For FAPbBr${}_{3}$ nanocrystals, 0.1 mmol lead bromide (36.7 mg) and 0.1 mmol formamidinium bromide (12.5 mg) were dissolved in 0.5 ml of DMF, forming the precursor solution. Separately, 10 μL of octylamine and 0.5 mL of oleic acid were added to 5 mL of toluene and mixed at high speed using a magnetic stirrer. 0.15 mL of the precursor solution was quickly added to the toluene solution, causing nanocrystals to precipitate immediately. The nanocrystal dispersion was centrifuged at 4750 RPM (4238 RCF) for 15 min, and the supernatant was discarded, so as to remove unreacted materials that were leftover from the synthesis. Using an ultrasonic bath, the remaining precipitate was dispersed in up to 5 mL of toluene and centrifuged for a second time, again at 4750 RPM for 15 min. The supernatant, which consisted of a colloidal suspension of small nanocrystals in toluene, was retained. The precipitate, which consisted of larger particles, was discarded. To produce FAPbCl${}_{3}$ nanocrystals, or mixed-halide nanocrystals, a portion of the lead bromide and the formamidinium bromide in the precursor solution were substituted for lead chloride and formamidinium chloride, respectively.

### 4.3. Surface Modification with BMEP

For a sample of FAPbBr${}_{3}$ nanocrystals prepared as above, 100 nL BMEP was added, and the mixture was left stirring in an ice bath for three hours.

### 4.4. Fabrication of EJ-290 Nanocomposites

The EJ-290 plastic scintillator (provided as a three-part kit) was prepared according to the instructions provided by Eljen Technology. After the three components had been combined, a concentrated dispersion of nanocrystals in toluene was added, and the mixture was placed in an ultrasonic bath. The nanocomposites were cast in PTFE moulds in a vacuum oven at 47 ${}^{\circ }$C for at least 14 days until solid and were then polished using silicon carbide paper and aluminium oxide powder.

### 4.5. Fabrication of PMMA Nanocomposites

1.67 g PMMA was added to 5 mL of toluene-dispersed nanocrystals. This mixture was heated and stirred for a period of several hours until the PMMA had fully dissolved. The solution was then poured into PTFE moulds, covered with a glass beaker, and left to harden overnight.

### 4.6. Calculations

Photoluminescence peaks were fit using pseudo-Voigt profiles. In this function, a Gaussian (G(λ, σ)) and a Cauchy–Lorentz (L(λ, γ)) distribution were convolved using a mixing parameter η and amplitude *A* [45]:

(1) V(λ)=A(ηG(λ,σ)+(1−η)L(λ,γ))

Mie scattering calculations were performed using the Python code from [46]. The refractive index of the FAPbBr${}_{3}$ nanocrystals was taken from the work of Ndione et al. [47].

### 4.7. Characterisation Methods

Photoluminescence spectra were obtained at room temperature using either a 20 mW 405 nm or a pulsed 355 nm laser and were acquired in the raw data mode of an OceanOptics QE65000 spectrometer. Time-resolved photoluminescence measurements were taken using a pulsed 405 nm laser and a PicoQuant fluorescence lifetime spectrometer (Picoquant, Berlin, Germany). For TEM images and for the EDS maps shown in Figure 2, nanocrystals dispersed in toluene were drop-cast onto holey carbon films on 300 Mesh copper grids. A Thermo Fisher Talos F200i microscope was used with an accelerating voltage of 200 keV. The EDS maps in Figure S4 in the Supporting Information were produced using a Jeol 7100F scanning electron microscope (Jeol, Tokyo, Japan), with an accelerating voltage of 15 keV. Dynamic light scattering (DLS) was performed using a Malvern Zetasizer Nanoseries (Malvern Panalytical, Malvern, UK) instrument in glass cuvettes; the results presented are an average of 15 measurements. Nanocrystal dispersions were sonicated before measurement in order to break up aggregates. Radioluminescence spectra and X-ray images were obtained using a Hamamatsu L6732-01 X-ray tube (Hamamatsu Photonics, Hamamatsu, Japan) at 80 kV, 100 μA. An OceanOptics QE65000 spectrometer was used to record the spectra, and an ELP webcam with a 2.8–12 mm varifocal lens was used to record the images. Optical transmission measurements were recorded using a Cary 5000 UV-vis spectrophotometer (Agilent, Santa Clara, CA, USA). For elemental lead analysis, samples were digested using microwave digestion systems (ETHOS UP, Analytix, Boldon, UK) using 5 mL of 37% HNO${}_{3}$ and diluted with ultrapure water to the required concentration. The elemental analysis of lead (λ = 405.781 nm) was carried out by microwave plasma atomic emission spectrometry (MP-AES 4200, Agilent, Santa Clara, CA, USA). Calibration solutions were prepared from commercially available spectroscopic standards of 1000 mg L${}^{-1}$ and diluted with 0.1 M HNO${}_{3}$. All data collected were corrected with a beryllium internal standard at 5 ppm (λ = 234.861 nm). Standards and sample solutions were introduced by an autosampler (ASX-500, Agilent, Santa Clara, CA, USA), with 60 s of rinsing between each. The pump speed was 15 rpm with uptake and stabilisation times of 40 s and 15 s, respectively. The nebulisation flow rate was 0.75 L/min for both Pb and Be.

## Figures and Tables

**Table 1:** Time constants for FAPbBr${}_{3}$ nanocomposite scintillators.

	$\tau_{1}$: Component from EJ-290	$\tau_{2}$: Component from FAPbBr${}_{3}$	$\tau_{3}$: Component from FAPbBr${}_{3}$	$\tau_{\mathrm{av}}$
EJ-290	1.2 ns (100%)	-	-	(1.2 ± 0.1) ns
FAPbBr${}_{3}$/EJ-290, 0.4% Loading	1.5 ns (44%)	17.5 ns (36%)	90.3 ns (20%)	(25.0 ± 0.1) ns
FAPbBr${}_{3}$/EJ-290, 0.9% Loading	1.8 ns (38%)	15.6 ns (43%)	75.1 ns (19%)	(21.8 ± 0.1) ns
FAPbBr${}_{3}$/EJ-290, 1.8% Loading	1.9 ns (36%)	14.9 ns (46%)	71.0 ns (18%)	(20.3 ± 0.1) ns
FAPbBr${}_{3}$/PMMA, 1.8% Loading	-	16.9 ns (56%)	119.7 ns (44%)	(62.0 ± 0.4) ns

## Data Availability

The data supporting the findings of this study are available by request to the corresponding author.

## Supplementary Materials

The following supporting information can be downloaded at: https://www.mdpi.com/article/10.3390/nano12132141/s1. Figure S1: When the size of a nanocrystal is reduced so as to approach the exciton Bohr radius for that material, quantum confinement effects cause the emission wavelength to become shorter. Here, the emission wavelength of various samples of FAPbBr_3_ nanocrystals (either that we have synthe-sised, or which have been reported in the literature [22,23,36,48,49,50,51,52,53]) are compared to the nanocrystal size (for our samples, this size is derived from the hydrodynamic radius, as measured by dynamic light scattering). The emission wavelengths are also compared to that of a polycrystalline film of FAPbBr_3_ [54]. Figure S2: Particle size distribution (hydrodynamic radius) for FAPbBr_3_ nanocrystals, measured by dynamic light scattering. Figure S3: TEM image of FAPbBr_3_ nanocrystals without BMEP-modification. Figure S4: Photos and EDS maps of nanocomposites containing large (>200 nm) FAPbBr_3_ nanocrystals. For nanocrystals of this size, a reduction in nanocrystal aggregation is clearly visible when the BMEP ligand is used. Figure S5: The Stokes shift of FAPbBr_3_ nanocrystals is small, and is also size-dependent: for nanocrystals with an average size of 19 nm (size derived from the hydrodynamic radius, as measured by dy-namic light scattering), the Stokes shift is 0.05 eV. For larger particles of FAPbBr_3_, the Stokes shift is even smaller, at 0.01 eV. Figure S6: Intensity calibration for microwave digestion of nanocomposite samples. This calibration was performed from standard samples with known quantities of lead (internal standard corrected). Table S1: Time constants for mixed halide FAPbBr_3x_Cl_3(1−x)_ nanocrystals in PMMA. Table S2: Microwave digestion results for FAPbBr_3_/EJ-290 composites, and for FAPbBr_3_/PMMA composite.
