# Supplementary material for: Formamidinium Lead Halide Perovskite Nanocomposite Scintillators
Source: Nanomaterials (Basel). 2022 Jun 22;12(13):2141. doi: 10.3390/nano12132141 (PMC9268382; doi:10.3390/nano12132141)
Supplement: Supplementary file 1 [file nanomaterials-12-02141-s001.zip › nanomaterials-1768515-supplementary.pdf]

nanomaterials
MDPI

Supporting Information

Formamidinium Lead Halide Perovskite Nanocomposite Scintillators

Isabel H. B. Braddock [1], Maya Al Sid Cheikh [2], Joydip Ghosh [1], Roma E. Mulholland [2], Joseph G. O'Neill [1], Vlad Stolojan [3], Carol Crean [2], Stephen J. Sweeney [1,3] and Paul J. Sellin [1,*]

[1] Department of Physics, University of Surrey, Guildford GU2 7XH, UK; i.braddock@surrey.ac.uk (I.H.B.B.); j.ghosh@surrey.ac.uk (J.G.); j.g.o'neill@surrey.ac.uk (J.G.O.); s.sweeney@surrey.ac.uk (S.J.S.)
[2] Department of Chemistry, University of Surrey, Guildford GU2 7XH, UK; m.alsidcheikh@surrey.ac.uk (M.A.S.C.); rm00849@surrey.ac.uk (R.E.M.); c.crean@surrey.ac.uk (C.C.)
[3] Advanced Technology Institute, University of Surrey, Guildford GU2 7XH, UK; v.stolojan@surrey.ac.uk (V.S.); s.sweeney@surrey.ac.uk (S.J.S.)
* Correspondence: p.sellin@surrey.ac.uk.

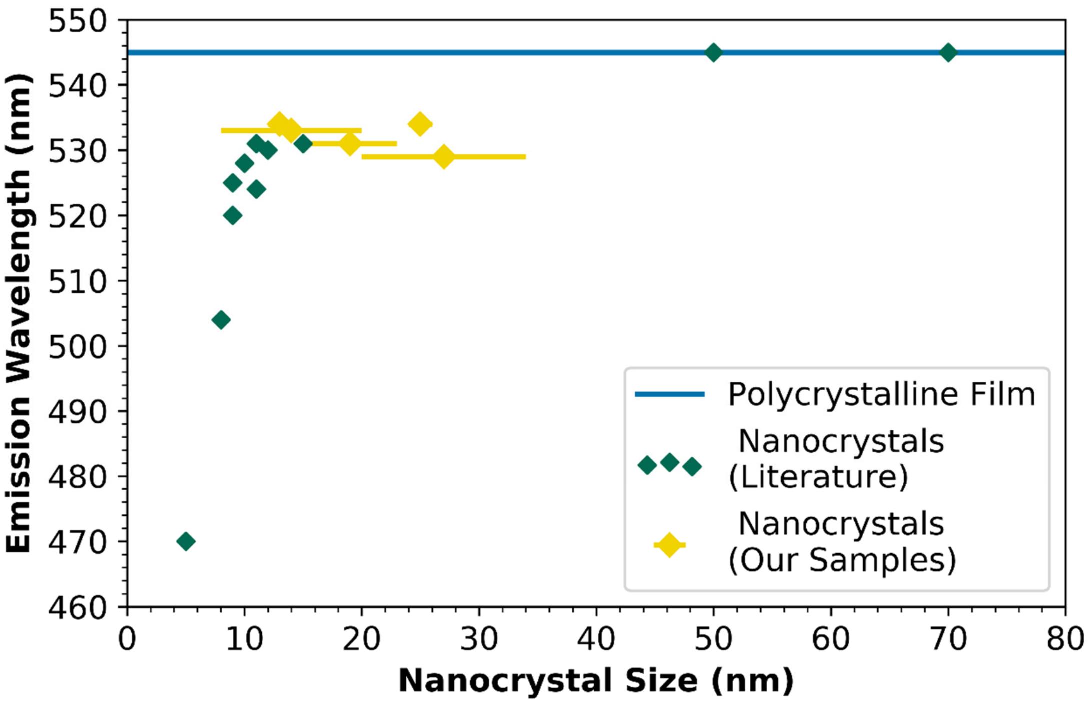

Figure S1. When the size of a nanocrystal is reduced so as to approach the exciton Bohr radius for that material, quantum confinement effects cause the emission wavelength to become shorter. Here, the emission wavelength of various samples of $FAPbBr_3$ nanocrystals (either that we have synthesised, or which have been reported in the literature [1–9]) are compared to the nanocrystal size (for our samples, this size is derived from the hydrodynamic radius, as measured by dynamic light scattering). The emission wavelengths are also compared to that of a polycrystalline film of $FAPbBr_3$ [10].

Table S1. Time constants for mixed halide $FAPbBr_{3x}Cl_{3(1-x)}$ nanocrystals in PMMA.

	τ_1 (ns)	τ_2 (ns)	τ_{av} (ns)
**$FAPbBr_3$**	16.9 (56%)	119.7 (44%)	62.0 ± 0.4
**$FAPbBr_{2.7}Cl_{0.3}$**	8.7 (67%)	39.8 (33%)	19.0 ± 0.2
**$FAPbBr_{2.4}Cl_{0.6}$**	9.7 (60%)	46.1 (40%)	24.2 ± 0.2
**$FAPbBr_{2.1}Cl_{0.9}$**	4.3 (53%)	20.1 (47%)	11.8 ± 0.1
**$FAPbBr_{1.8}Cl_{1.2}$**	2.7 (52%)	18.9 (48%)	10.4 ± 0.1
**$FAPbBr_{1.5}Cl_{1.5}$**	3.9 (53%)	29.6 (47%)	15.9 ± 0.1
**$FAPbBr_{1.2}Cl_{1.8}$**	1.6 (62%)	15.3 (38%)	6.8 ± 0.1
**$FAPbBr_{0.9}Cl_{2.1}$**	1.2 (66%)	20.9 (34%)	7.8 ± 0.1
**$FAPbBr_{0.6}Cl_{2.4}$**	1.1 (67%)	20.1 (33%)	7.3 ± 0.1

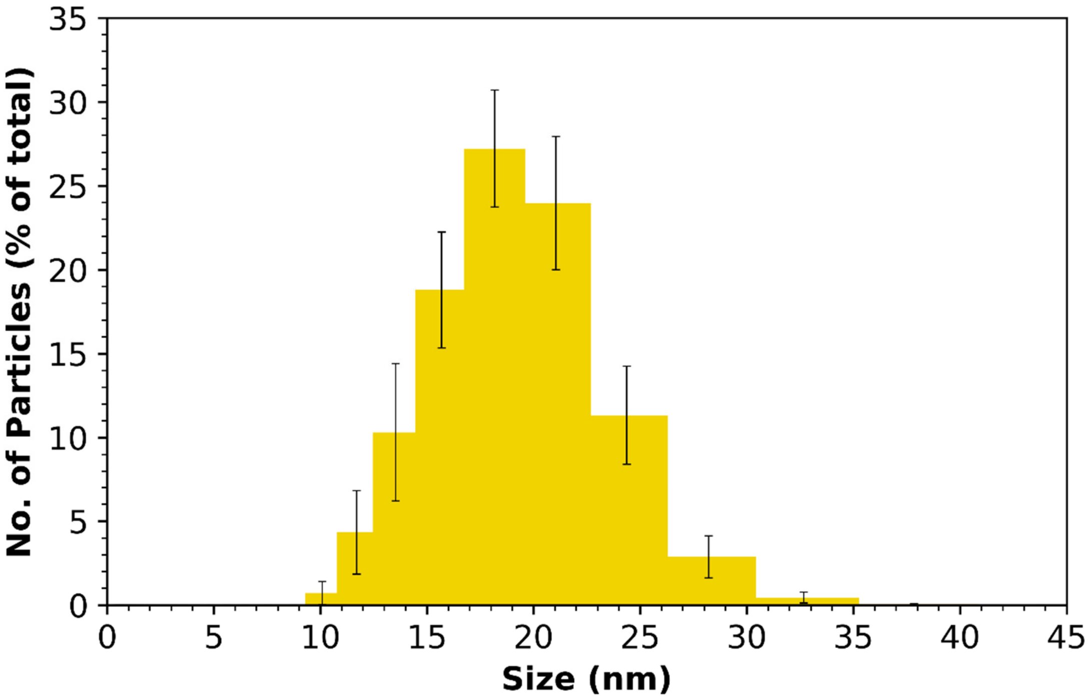

Figure S2. Particle size distribution (hydrodynamic radius) for $FAPbBr_3$ nanocrystals, measured by dynamic light scattering.

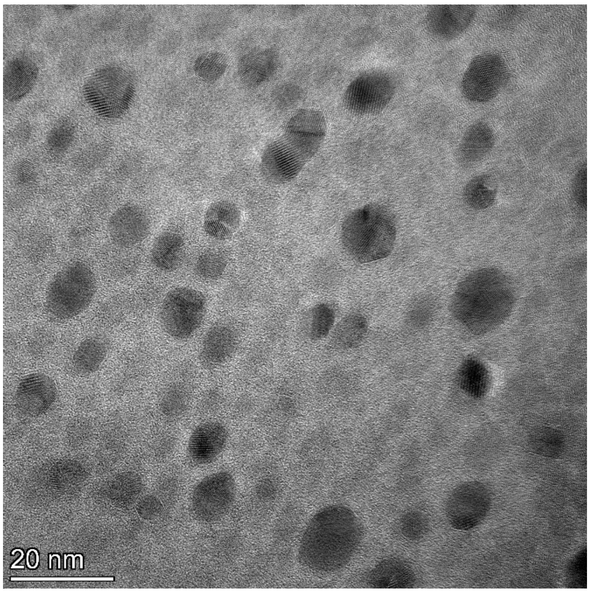

Figure S3. TEM image of $FAPbBr_3$ nanocrystals without BMEP-modification.

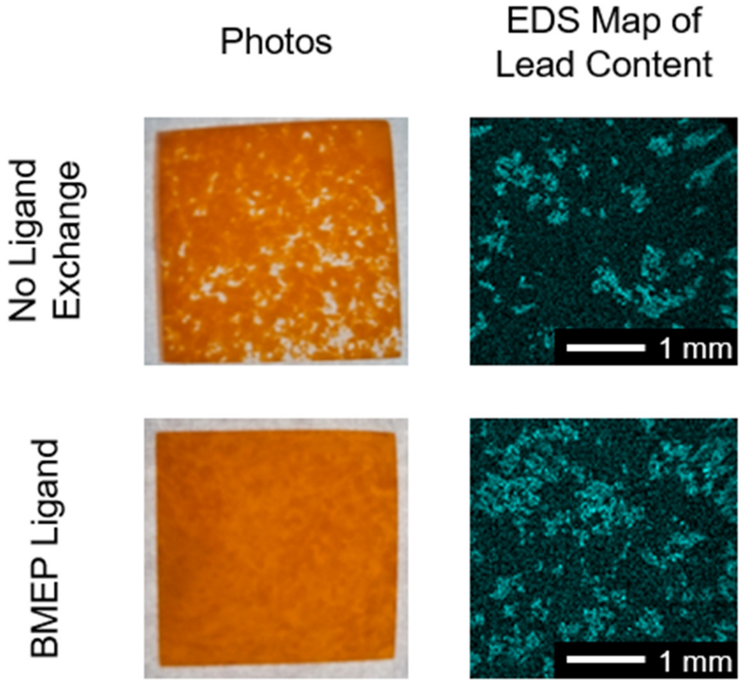

Figure S4. Photos and EDS maps of nanocomposites containing large (>200 nm) $FAPbBr_3$ nanocrystals. For nanocrystals of this size, a reduction in nanocrystal aggregation is clearly visible when the BMEP ligand is used.

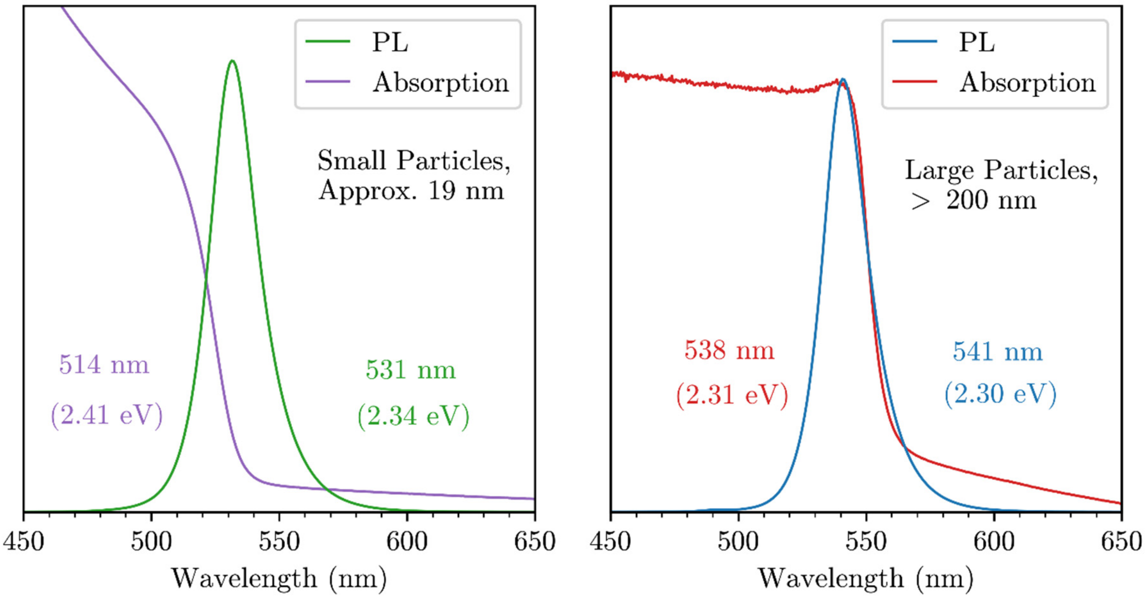

Figure S5. The Stokes shift of $FAPbBr_3$ nanocrystals is small, and is also size-dependent: for nanocrystals with an average size of 19 nm (size derived from the hydrodynamic radius, as measured by dynamic light scattering), the Stokes shift is 0.05 eV. For larger particles of $FAPbBr_3$, the Stokes shift is even smaller, at 0.01 eV.

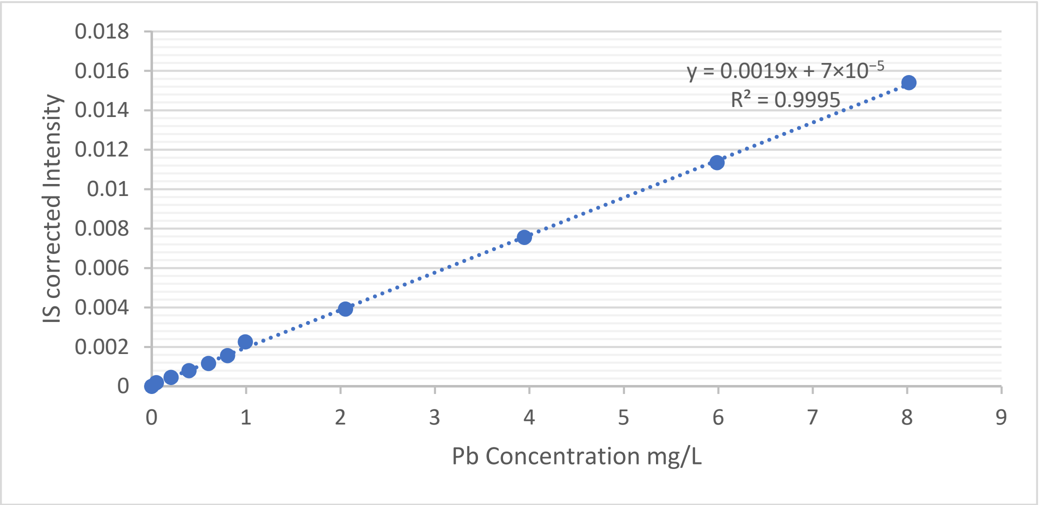

Figure S6. Intensity calibration for microwave digestion of nanocomposite samples. This calibration was performed from standard samples with known quantities of lead (internal standard corrected).

Table S2. Microwave digestion results for $FAPbBr_3$/EJ-290 composites, and for $FAPbBr_3$/PMMA composite.

Sample	Intensity	Lead Content (ppm)	Sample Mass (g)	% Lead in Sample
$FAPbBr_3$/PMMA composite	0.008715	4.5469	0.0142	0.94%
$FAPbBr_3$/EJ-290 Composite 1	0.001382	0.6904	0.0102	0.20%
$FAPbBr_3$/EJ-290 Composite 2	0.001918	0.9723	0.0539	0.38%

The loading of $FAPbBr_3$/EJ-290 Composite 3 was extrapolated from these results as 0.78%, as the loading was known to be double that of $FAPbBr_3$/EJ-290 Composite 2, and four times that of $FAPbBr_3$/EJ-290 Composite 1.

References

1. Protesescu, L.; Yakunin, S.; Bodnarchuk, M.I.; Bertolotti, F.; Masciocchi, N.; Guagliardi, A.; Kovalenko, M.V. Monodisperse Formamidinium Lead Bromide Nanocrystals with Bright and Stable Green Photoluminescence. *J. Am. Chem. Soc.*, **2016**, *43*, 14202–14205.
2. Fan, L.; Ding, K.; Chen, H.; Xiang, S.; Zhang, R.; Guo, R.; Liu, Z.; Wang, L. Efficient pure green light-emitting diodes based on formamidinium lead bromide perovskite nanocrystals. *Organic Electronics*, **2018**, *60*, 64–70.
3. Tamarat, P.; Bodnarchuk, M.I.; Trebbia, J-B.; Erni, R.; Kovalenko, M.V.; Even, J.; Lounis, B. The ground exciton state of formamidinium lead bromide perovskite nanocrystals is a singlet dark state. *Nature Materials*, **2019**, *18*, 717–724.
4. Kim, Y.H.; Lee, G.H.; Kim, Y.T.; Wolf, C.; Yun, H.J.; Kwon, W.; Park, C.G.; Lee, T.-W. High efficiency perovskite light-emitting diodes of ligand-engineered colloidal formamidinium lead bromide nanoparticles. *Nano Energy*, **2017**, *38*, 51–58.
5. Minh, D.N.; Kim, J.; Hyon, J.; Sim, J.H.; Sowlih, H.H.; Seo, C.; Nam, J.; Eom, S.; Suk, S.; Lee, S.; Kim, E.; Kang, Y. Room-Temperature Synthesis of Widely Tunable Formamidinium Lead Halide Perovskite Nanocrystals. *Chemistry of Materials*, **2017**, *29*, 5713–5719.
6. Trinh, C.T.; Minh, D.N.; Ahn, K.J.; Kang, Y.; Lee, K.-G. Organic–Inorganic $FAPbBr_3$ Perovskite Quantum Dots as a Quantum Light Source: Single-Photon Emission and Blinking Behaviors. *ACS Photonics*, **2018**, *5*, 4937–4943.
7. Perumal, A.; Shendre, S.; Li, M.; Tay, Y.K.E.; Sharma, V.K.; Chen, S.; Wei, Z.; Liu, Q.; Gao, Y.; Buenconsejo, P.J.S.; Tan, S.T.; Gan, C.L.; Xiong, Q.; Sum, T.C.; Demir, H.V. High brightness formamidinium lead bromide perovskite nanocrystal light emitting devices. *Scientific Reports*, **2016**, *6*, 36733
8. Aygüler, M.F.; Weber, M.D.; Puscher, B.M.D.; Medina, D.D.; Docampo, P.; Costa, R.D. Light-emitting electrochemical cells based on hybrid lead halide perovskite nanoparticles. *J. Phys. Chem. C*, **2015**, *119*, 12047
9. Jana, A.; Mittal, M.; Singla, A.; Sapra, S. Solvent-free, mechanochemical syntheses of bulk trihalide perovskites and their nanoparticles. *Chem. Commun.*, **2017** ,*53*, 3046–3049
10. Eperon, G.; Stranks, S.D.; Menelaou, C.; Johnston, M.B.; Herz, L.M.; Snaith, H.J. Formamidinium lead trihalide: a broadly tunable perovskite for efficient planar heterojunction solar cells. *Energy Environ. Sci.*, **2014**, *7*, 982–988
